# Tenth anniversary of the Young DEGRO Team Trial—reflecting on the past, shaping the future

**DOI:** 10.1007/s00066-025-02417-9

**Published:** 2025-06-24

**Authors:** Felix Ehret, Michael Oertel, Alexander Rühle

**Affiliations:** 1 Department of Radiation Oncology, Charité – Universitätsmedizin Berlin, Corporate Member of Freie Universität Berlin and Humboldt-Universität zu Berlin, Berlin, Germany; 2 German Cancer Consortium (DKTK), partner site Berlin, a partnership between DKFZ and Charité – Universitätsmedizin Berlin, Berlin, Germany; 3 Young DEGRO Team Trial, DEGRO, Berlin, Germany; 4 Department of Radiation Oncology, University Hospital Münster, Münster, Germany; 5 West German Cancer Center Münster (WTZ), Münster, Germany; 6 Department of Radiation Oncology, University of Leipzig Medical Center, Leipzig, Germany; 7 Comprehensive Cancer Center Central Germany, Partner Site Leipzig, Leipzig, Germany; 8 Department of Radiation Oncology, Medical Center – University of Freiburg, Faculty of Medicine, University of Freiburg, German Cancer Consortium (DKTK), partner site DKTK-Freiburg, Freiburg, Germany

The Team Trial of the workgroup Young German Society of Radiation Oncology (DEGRO) commemorates its 10th anniversary in 2025. The following editorial summarizes past developments, highlights successes, and reflects on future objectives.

The Young DEGRO is a dedicated workgroup of the DEGRO with the goal of supporting and connecting young radiation oncologists, biologists, and physicists during their training. From the beginning, one of the primary purposes of the Young DEGRO was to connect committed young colleagues at different institutions to establish collaborations in clinical practice, teaching, and research. The latter was driven by the idea of creating a platform to realize one’s own research ideas, lower barriers to engagement, and strengthen scientific cooperation within the young radiation oncology community. These ideas align with insights from a survey by the Young DEGRO, revealing a broad interest in research [1]. Therefore, creating a dedicated subgroup or team within the Young DEGRO to foster research was a logical consequence.

One fundamental principle of the Team Trial is that all research, even when conducted by junior scientists, must adhere to established ethical guidelines and follow state-of-the-art methodology. Consequently, high quality standards have been implemented to select suitable study proposals. The selection process for upcoming studies has evolved into a structured, peer-reviewed system that considers originality and benefits for the workgroup and community as well as feasibility regarding study execution and analysis. It became evident that effective planning and coordination of studies requires a decisive leadership role within the Young DEGRO, now known as the Speaker of the Team Trial.

The scientific collaborations of the Team Trial now extend beyond the community of young researchers to include other workgroups, such as the DEGRO workgroup Radiosurgery and Stereotactic Radiotherapy and the workgroup Radiological Oncology of the German Cancer Society (ARO) [2]. The partnership with ARO, in particular, has developed into a productive collaboration that fosters the research ideas of young colleagues. Additionally, several studies by the Team Trial have been recognized as official ARO studies or honored with ARO awards, further demonstrating the high scientific standards upheld by the workgroup [3].

Overall, nine main studies have been or are currently being conducted by the Team Trial, many of which have led to secondary analyses and multiple publications (Table 1). These studies have resulted in numerous contributions to national and international journals and conferences, marking considerable success within the young radiation oncology community and beyond. The following three representative studies provide a brief glimpse into some of these achievements.

**Table 1:** Overview of previous and current studies of the Young DEGRO Team Trial

Study title/topic	Short title	ARO label	Principal investigator(s)	Study type	No. patients/participants	No. centers	Main publications
Prognostic Evaluation of Tumor Volume and its Changes in Radical Radiotherapy of Advanced NSCLC (NCT03055715)	NA	2017-01	Christian Ostheimer	International multicenter retrospective cohort study	347	21	[13, 14]
Reirradiation for Recurrent Head and Neck Cancer	HNC Re-RT	2022-05	Johannes Roesch, Markus Hecht	Multicenter retrospective cohort study	253	16	[15]
Neuroblastoma: Local Control and Recurrence Patterns After Radiotherapy for High-Risk Patients in the NB97 and NB2004HR Trials	NA	NA	Danny Jazmati	Post-hoc analysis of two prospective multicenter trials	Recruitment completed, final analysis pending		
Financial Toxicity in Cancer Patients Treated with Radiotherapy	FinTox	2022-07	Alexander Fabian	Multicenter, noninterventional, cross-sectional study	1075	11	[5–7]
Abscopal Effects in Metastatic Cancer Patients Under Radioimmunotherapy	ARTIC	2022-10	Maike Trommer, Simone Ferdinandus	Multicenter retrospective cohort study	Recruitment completed, final analysis completed, manuscript in preparation		
Outcome and Toxicity of Total Neoadjuvant Therapy in Rectal Cancer	TNTox	2023-12	Georg Wurschi	Multicenter retrospective cohort study	Recruitment completed, final analysis pending		
Survey on the Current Situation of Young Biologists, Medical Physicists, and Physicians in Radiation Research	NA	NA	Annemarie Schröder, Lisa Deloch, Thomas Weissmann	Web-based survey	218	NA	[8]
Stereotactic Body Radiotherapy for Pulmonary Metastases in Patients with Oligometastatic Head and Neck Squamous Cell Carcinoma	SBRT OligoLuMet HNSCC	NA	Alexander Rühle	International multicenter retrospective cohort study	223 (178 at the time of the first analysis)	17 (16 at the time of the first analysis)	[4]
Medial Retropharyngeal Lymph Involvement in Non-Endemic Nasopharyngeal Carcinoma	MERLIN	NA	Justus Kaufmann	Multicenter retrospective cohort study	Recruiting		

*ARO* workgroup Radiological Oncology of the German Cancer Society, *No.* number, *NA* not available, *NSCLC* non-small cell lung cancer

## Representative studies

### Stereotactic body radiotherapy for pulmonary metastases in patients with oligometastatic head and neck squamous cell carcinoma (SBRT OligoLuMet HNSCC)

**Fig. 1:**
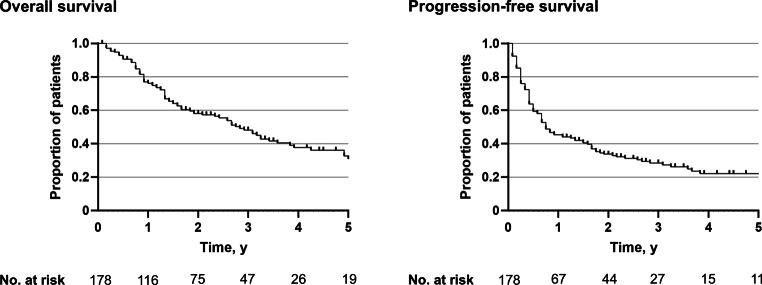
Overall survival and progression-free survival in patients with oligometastatic head and neck squamous cell carcinoma (*HNSCC*) treated with stereotactic body radiotherapy (*SBRT*) for lung metastases. The median follow-up time was 40 months [4]. (Reprinted with approval of the publisher)

The SBRT OligoLuMet HNSCC study was a multicenter cohort study that evaluated the outcomes of stereotactic body radiotherapy (SBRT) for pulmonary metastases in patients with oligometastatic head and neck squamous cell carcinoma (HNSCC). Conducted across 16 centers in Germany, Austria, and Switzerland, the study retrospectively included 178 patients with 284 irradiated lung metastases treated between 2010 and 2023 [4]. The median overall survival was 33 months, while the median progression-free survival was 9 months (Fig. 1). The 1‑year cumulative incidence of local failures was 5.5%, indicating excellent local control. Toxicity was minimal, with only one patient experiencing grade 3 acute toxicity and two patients developing chronic grade 3 toxicities, while no grade 4 or 5 toxicities were reported. Older age and female sex were associated with worse overall survival, whereas a longer time between the HNSCC diagnosis and SBRT was linked to improved survival. The findings suggest that SBRT is a safe and effective treatment option for pulmonary metastases in oligometastatic HNSCC. However, prospective trials are needed to determine the optimal integration of SBRT with systemic therapies.

A distinctive feature of this study is its collaboration between the Young DEGRO Team Trial and the DEGRO workgroup Radiosurgery and Stereotactic Radiotherapy, leveraging their combined expertise and resources. While the primary results have been published in the *Red Journal*, a secondary analysis focusing on tumor control probability models is in preparation. One of the study’s key strengths is its large patient cohort, making it the most comprehensive investigation on this topic to date.

### Financial toxicity in cancer patients treated with radiotherapy (FinTox)

The FinTox study was a prospective, multicenter, noninterventional, confirmatory, cross-sectional study that evaluated financial toxicity in cancer patients undergoing radiotherapy within a universal healthcare system. Conducted across 11 centers in Germany, the study surveyed 1075 patients over a 60-day period, assessing their subjective financial distress using the European Organisation for Research and Treatment of Cancer (EORTC) QLQ-C30 questionnaire. The study was preregistered at the German Clinical Trial Registry (DRKS00028784) and received an official label from the ARO (ARO 2022-07).

**Fig. 2:**
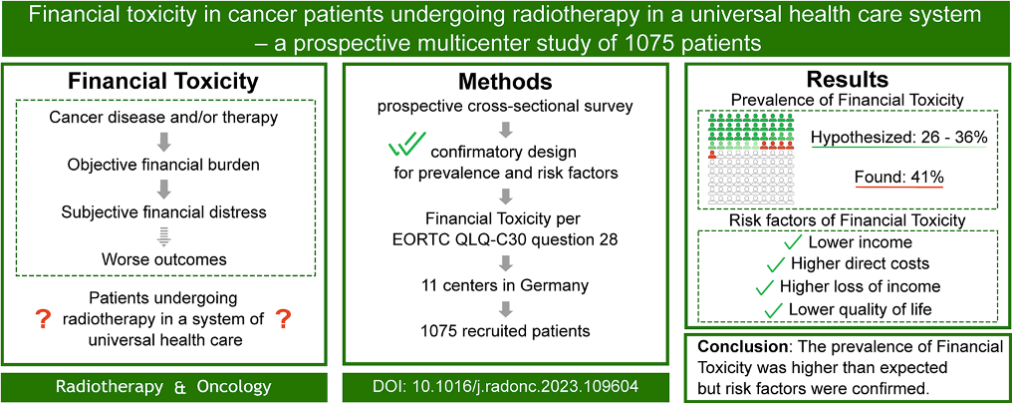
This prospective cross-sectional study assessed financial toxicity in cancer patients undergoing radiotherapy across 11 centers in Germany over 60 days using the European Organisation for Research and Treatment of Cancer (EORTC) QLQ-C30 financial distress item as a surrogate measure. Among 2341 eligible patients, 1075 (46%) participated, with 41% reporting subjective financial distress, exceeding the expected prevalence range [5]. (Reprinted with approval of the publisher)

The study found that 41% of patients experienced financial distress, exceeding the expected range of 26–36% [5]. Financial toxicity was rated as “a little” by 26%, “quite a bit” by 11%, and “very much” by 4% (Fig. 2). The primary risk factors associated with increased financial distress included lower household income, higher direct costs (e.g., treatment-related expenses), loss of income, and lower overall health-related quality of life. Additionally, higher psychosocial distress and lower patient satisfaction were also significantly associated with increased financial toxicity.

Financial toxicity was more prevalent than anticipated, even in a universal healthcare setting, suggesting that financial burden remains a significant concern for cancer patients. The study highlights the need for early identification of at-risk patients and support strategies to mitigate financial toxicity.

Two secondary analyses were conducted based on data from this trial. The first secondary analysis examined psychosocial distress among the 1042 patients who completed the distress thermometer [6]. Applying a cutoff of ≥ 5, the study found that 63% of patients experienced clinically significant distress at the end of radiotherapy. Key risk factors for increased distress included lower education level, greater income loss, reduced quality of life, and a longer radiotherapy duration.

The other secondary analysis focused on patient satisfaction with radiotherapy care among cancer patients in Germany [7]. Of 1054 patient-reported responses, 85% expressed high satisfaction (≥ 8 on a 10-point Likert scale). Factors associated with lower satisfaction included rectal cancer as a tumor entity, inpatient care, concomitant chemotherapy, treatment center, lower income, higher costs, and reduced quality of life. Multivariable analysis confirmed that tumor type, treatment center, and quality of life were key determinants of patient satisfaction, providing valuable insights for optimizing patient-centered care in radiotherapy.

This study exemplifies the value of multicenter studies within the Young DEGRO Team Trial network, demonstrating how collaborative efforts can drive rapid patient recruitment. With over 1000 patients enrolled in just 60 days, it stands as one of the fastest-recruiting studies in the radiation oncology community.

### Survey on the current situation of young biologists, medical physicists, and physicians in radiation research

Experimental, translational, and clinical research are vital to advancing radiation oncology. A fundamental requirement for achieving this objective is to recruit and retain qualified personnel, especially young professionals. However, with ever-changing working conditions, demographic changes, and persistent economic pressure in healthcare systems, it is essential to identify the needs of the next generation of young researchers to ensure long-term success in radiation research. Therefore, a survey study on the current situation of young biologists, medical physicists, and physicians in radiation research was done.

The study was conducted in the Young DEGRO network in close collaboration with the German Society for Biological Radiation Research (DeGBS) and the German Society for Medical Physics (DGMP) [8]. The web-based survey included a general part with 13 questions and between 20 and 25 specialty-specific questions. It was distributed for 6 weeks, from June to August 2023. The questions aimed to identify the current situation and needs of young professionals in radiation oncology and radiation research.

Nearly 220 participants were registered, including 59 biologists, 70 physicists, and 89 physicians. The results of the survey not only showed general agreement on specific topics but also distinct differences between specialties, highlighting the necessity of providing individualized solutions for each profession. For instance, replies from biologists demonstrated the persistent challenges to securing long-term funding and attractive job positions. Notably, a considerable proportion of biologists indicate that they might leave radiation research in the future, thereby underscoring the need for improvements. Conversely, the surveyed physicists reported a high satisfaction with their career choice. They also indicated the highest percentage of permanent contracts among all professions. Interestingly, they also showed the most pronounced willingness to leave academia, highlighting the potential need for academic institutions to prepare accordingly. Finally, concerns raised by young physicians included significant economic pressure, lack of work–life balance, and the compatibility of career and family. Among all specialties, physicians indicated the highest subjective workload. This is aggravated by the fact that research is often conducted outside regular working hours, calling for more protected research time and additional clinician-scientist programs.

In summary, this contemporary survey study spearheaded by the Young DEGRO Team Trial provided comprehensive data and revealed valuable insights into the current needs of young professionals in the field. These insights can help to address the most pressing challenges, in order to retain young biologists, physicists, and physicians in radiation research. The study underscores the objective and dedication of the Young DEGRO Team Trial to address the needs of its members and ensure adequate support and recruitment of young professionals in the field.

## Summary and outlook

Since its foundation 10 years ago, the Young DEGRO Team Trial has established itself as a central platform within the DEGRO for young biologists, physicists, and physicians to plan, discuss, and conduct studies. Numerous publications, such as the three representative studies highlighted herein, showcase the success of the group over the past decade, fueled by the passion, dedication, and prowess of its members, with many of them now being attendings and principal investigators at various academic institutions throughout Germany. While we cherish and gratefully acknowledge the successes of the past, we want to shift our focus to the future.

Given the recent advances in radiation techniques, cancer biology, imaging, and artificial intelligence, radiation oncology research is of utmost importance to improve the outcomes of affected patients. While clinician-scientists are needed more than ever before, interdisciplinary research and strong collaborations among all involved professions and societies in radiation oncology will be necessary to ensure widespread access to and best use of radiotherapy [9–11].

The Young DEGRO Team Trial aims to contribute to these needs and foster the exchange and cooperation among young professionals in the coming years. With its strong foundation, we are eager to extend our research portfolio and group, implementing more studies and diverse projects, especially in medical physics and radiobiology, as well as welcoming and supporting new members. By doing so, we are actively contributing to the vision of the DEGRO for radiation oncology in Germany in 2030 – “Innovative radiation oncology Together – Precise, Personalized, Human” [12].
